# Over 19.2% Efficiency of Organic Solar Cells Enabled by Precisely Tuning the Charge Transfer State Via Donor Alloy Strategy

**DOI:** 10.1002/advs.202203606

**Published:** 2022-08-23

**Authors:** Jinhua Gao, Na Yu, Zhihao Chen, Yanan Wei, Congqi Li, Tianhua Liu, Xiaobin Gu, Jianqi Zhang, Zhixiang Wei, Zheng Tang, Xiaotao Hao, Fujun Zhang, Xin Zhang, Hui Huang

**Affiliations:** ^1^ College of Materials Science and Opto‐Electronic Technology Center of Materials Science and Optoelectronics Engineering CAS Center for Excellence in Topological Quantum Computation CAS Key Laboratory of Vacuum Physic University of Chinese Academy of Sciences Beijing 100049 China; ^2^ Center for Advanced Low‐Dimension Materials State Key Laboratory for Modification of Chemical Fibers and Polymer Materials College of Materials Science and Engineering Donghua University Shanghai 201620 China; ^3^ School of Physics State Key Laboratory of Crystal Materials Shandong University Jinan Shandong 250100 China; ^4^ Center for Excellence in Nanoscience (CAS) Key Laboratory of Nanosystem and Hierarchical Fabrication (CAS) National Center for Nanoscience and Technology Beijing 100190 China; ^5^ Key Laboratory of Luminescence and Optical Information Ministry of Education Beijing Jiaotong University Beijing 100044 China

**Keywords:** charge transfer state, donor alloy strategy, energy loss, organic solar cells

## Abstract

The large energy loss (*E*_loss_) is one of the main obstacles to further improve the photovoltaic performance of organic solar cells (OSCs), which is closely related to the charge transfer (CT) state. Herein, ternary donor alloy strategy is used to precisely tune the energy of CT state (*E*_CT_) and thus the *E*_loss_ for boosting the efficiency of OSCs. The elevated *E*_CT_ in the ternary OSCs reduce the energy loss for charge generation (Δ*E*_CT_), and promote the hybridization between localized excitation state and CT state to reduce the nonradiative energy loss (Δ*E*_nonrad_). Together with the optimal morphology, the ternary OSCs afford an impressive power conversion efficiency of 19.22% with a significantly improved open‐circuit voltage (*V*_oc_) of 0.910 V without sacrificing short‐cicuit density (*J*_sc_) and fill factor (FF) in comparison to the binary ones. This contribution reveals that precisely tuning the *E*_CT_ via donor alloy strategy is an efficient way to minimize *E*_loss_ and improve the photovoltaic performance of OSCs.

## Graphical abstract

Donor alloy strategy is proposed to tune chare transfer (CT) state erergy and thus *E*_loss_ for boosting organic solar cells (OSCs) efficiency. Together with optimal morphology, ternary OSCs deliver an outstanding efficiency of 19.22% with significantly improved open‐circuit voltage (*V*_oc_) of 0.910 V, the highest value for over 19% efficiency OSCs.

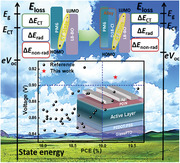

## 1 Introduction

In the past decades, organic solar cells (OSCs) have attracted substantial attention due to their merits of light weight, large‐area solution processability, and flexibility.^[^ ^1^, ^2^, ^3^, ^4^ ^]^ The power conversion efficiency (PCE) of OSCs has witnessed rapid progress with the development of donor and acceptor materials, device engineering, and in‐depth mechanism investigations.^[^ ^5^, ^6^, ^7^, ^8^ ^]^ However, the PCE of state‐of‐the‐art OSCs still lags behind those of nonexcitonic solar cells, such as silicon and perovskite solar cells.^[^ ^9^, ^10^, ^11^ ^]^ One of the most important factors limiting the PCE of OSCs is their large energy loss (*E*_loss_), resulting in a relatively low open circuit voltage (*V*_oc_) value, generally less than 0.9 V.^[^ ^12^, ^13^, ^14^ ^]^ Therefore, it is critical to reduce the *E*_loss_ to improve the *V*_oc_ and PCE of OSCs.^[^ ^15^, ^16^ ^]^

Unlike nonexcitonic solar cells, the charge transfer (CT) state exists in the excitonic OSCs due to the small dielectric constant and the localized electronic states of organic materials, thus leading to an additional energy loss channel (Δ*E*_CT_) and large nonradiative energy loss (Δ*E*_nonrad_).^[^ ^17^, ^18^, ^19^ ^]^ The Δ*E*_CT_ is considered as the energy loss for charge generation caused by the essential driving force for exciton dissociation in OSCs, which can be calculated using *E*_g_ − *E*_CT_, where *E*_g_ is the optical bandgap of the donor/acceptor materials, and *E*_CT_ is the energy of CT state. Apparently, increasing the *E*_CT_ by minimizing the energetic offset between the donor and the acceptor can effectively reduce Δ*E*_CT_.^[^ ^20^, ^21^ ^]^ Importantly, the reduced Δ*E*_CT_ can further promote the hybridization between the highly luminescent localized excitation (LE) state and CT state, which may increase the luminescence of CT state through an intensity borrowing mechanism, resulting in the decrease of Δ*E*_nonrad_.^[^ ^22^, ^23^ ^]^ However, the Δ*E*_CT_ reduction of OSCs may increase the risk of insufficient exciton dissociation and charge generation, resulting in an unsatisfactory short‐circuit current density (*J*_sc_).^[^ ^24^, ^25^ ^]^ Hence, precisely tuning the *E*_CT_ of OSCs is a crucial yet challenging issue.

Ternary OSCs with an alloy model were efficient methods to tune the *E*_CT_ to boost the photovoltaic performance.^[^ ^26^, ^27^ ^]^ The energy levels of the nonfullerene acceptor alloys can be tuned by varying its composition ratio, thus regulating the *E*_CT_, *E*_loss_, and *V*_oc_ of the corresponding OSCs.^[^ ^28^, ^29^ ^]^ The increased lowest unoccupied molecular orbital level of the acceptor alloys will be accompanied by the improvement of *E*_CT_ of the corresponding devices, resulting in the decrease of *E*_loss_ and the increase of *V*_oc_. However, the absorption spectra of the acceptor alloys will generally undergo a blueshift, which will narrow the photon harvesting range of the active layer, detrimental to the *J*_sc_ of OSCs.^[^ ^30^, ^31^ ^]^ To solve this trade‐off issue, donor alloys have been used to reduce the *E*_loss_ and increase the *V*_oc_ by incorporating a donor with a wide bandgap and deep highest occupied molecular orbital (HOMO) energy level as the third component. Simultaneously, the *J*_sc_ of OSCs may be increased by incorporating a wide bandgap donor into the active layers, which broadened the absorption range and thus improved its photon capture ability. However, the construction of ternary OSCs based on donor alloys remains great challenges due to the finite entropy of polymers, resulting in large repulsive intermolecular interaction between two polymer donors.^[^ ^32^, ^33^ ^]^ Very recently, a representative example of the ternary OSC based on two polymer donors provided a record high PCEs of over 19.0% by forming a double‐fibril network morphology in active layer.^[^ ^34^ ^]^ Thus, tuning *E*_CT_ via donor alloy, thereby increasing both *V*_oc_ and *J*_sc_, may be another promising strategy to improve photovoltaic performance.

Herein, the polymer donor D18‐Cl, possessing similar chemical structure and deeper HOMO energy level relative to PM6, was elaborately selected as the third component for the PM6:L8‐BO host system to fabricate efficient ternary OSCs. Impressively, PM6 and D18‐Cl formed donor alloy in ternary active layers due to their excellent miscibility, supported by differential scanning calorimetry (DSC) and cyclic voltammetry (CV) measurement. Accordingly, the energy levels and *E*_CT_ of the ternary system was tuned via simply varying D18‐Cl content, narrowing the gap between *E*_g_ and *E*_CT_. Thus, both Δ*E*_CT_ and Δ*E*_nonrad_ were reduced, resulting in a significantly increased *V*_oc_. Meanwhile, the incorporation of D18‐Cl broadens the optical absorption range and optimizes the morphology of the ternary film, affording enhanced short‐cicuit density (*J*_sc_) and fill factor (FF). Ultimately, the ternary OSCs delivered an outstanding PCE of 19.22% (certified PCE of 18.8%) with all‐around enhancement of *J*_sc_, *V*_oc_, and FF. The success of fine tuning the CT state was exemplified by the significantly improved *V*_oc_ of 0.910 V, the highest value for the OSCs with over 19% efficiency.

## 2 Results and Discussion

**Figure 1:**
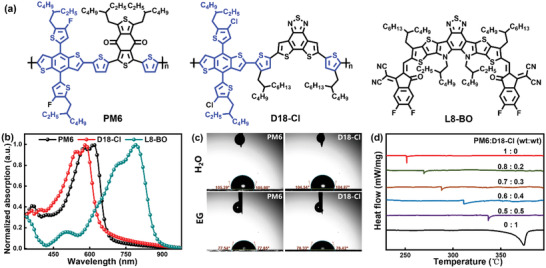
a) Chemical structures of PM6, D18‐Cl and L8‐BO. b) Normalized absorption spectra of neat films. c) Contact angle images of neat PM6 and D18‐Cl films. d) DSC curves of PM6:D18‐Cl blend with various D18‐Cl contents in the heating process.

The donor D18‐Cl was selected as the third component since it possesses similar chemical structure and deeper HOMO energy level relative to PM6. The chemical structures and energy levels of PM6, D18‐Cl, and L8‐BO are shown in **Figure** **1**a; and Figure S1 (Supporting Information). The normalized absorption spectra of these materials (Figure 1b) showed that the absorption of D18‐Cl is well complementary to those of PM6 and L8‐BO, which can broaden the optical absorption of the ternary blend film in comparison to the PM6‐based blend film (Figure S1, Supporting Information). Considering the high similarity of chemical structure of PM6 and D18‐Cl, the two polymer donors may have good compatibility to form an alloy like phase in ternary blend films.^[^ ^35^ ^]^ To verify the formation of the alloy states between PM6 and D18‐Cl, contact angle measurements were peformed to investigate the compatibility between two materials (Figure 1c; and Figure S2, Supporting Information). The surface tension (*γ*) of PM6 and D18‐Cl were calculated to be 23.92 and 24.21 mN m^−1^, respectively, as shown in Table S1 (Supporting Information). The miscibility of PM6 and D18‐Cl can be evaluated by the interfacial energy between the two materials, which can be calculated according to their surface energy.^[^ ^36^ ^]^ The calculated interfacial energy of PM6:D18‐Cl blend is 0.02 mN m^−1^, which is much smaller than those of PM6/L8‐BO and D18‐Cl/L8‐BO, indicating that the two materials are fully miscible in ternary blend films to form a donor alloy. In addition, DSC measurements were carried out to investigate the changes of melting behavior of PM6:D18‐Cl blend with various D18‐Cl contents. As shown in Figure 1d, the melting peaks appear at 251.6 and 374.2 °C for PM6 and D18‐Cl, respectively, in the process of heating. After mixing D18‐Cl with PM6 with different weight ratio, the melting peak of the PM6:D18‐Cl mixture moved from 269.7 °C (0.8:0.2) to 288.2 °C (0.7:0.3), and then to 311.6 °C (0.6:0.4) and 337.1 °C (0.5:0.5), which exhibited composition ratio dependence, indicating that PM6 and D18‐Cl can be well mixed to form alloy phases.^[^ ^37^ ^]^ Besides, CV measurement showed that the HOMO energy levels of PM6:D18‐Cl blends gradually decreased with increasing of D18‐Cl content, which is considered as a common feature of forming alloy state (Figure S1, Supporting Information).^[^ ^38^ ^]^ Therefore, the formation of PM6:D18‐Cl alloy can be well confirmed, which is conducive to tune the energy level of the donor in active layer over a wide range of D18‐Cl contents without generating charge traps. It is thus beneficial to precisely tune the *E*_CT_ of ternary OSCs.^[^ ^39^, ^40^ ^]^

**Figure 2:**
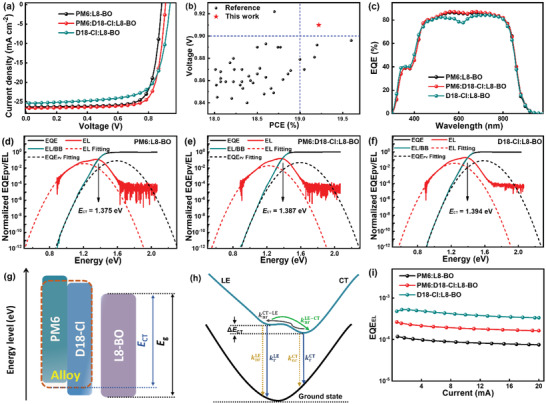
a) *J–V* curves of the binary and ternary OSCs. b) The *V*_oc_ values of efficient OSCs with PCEs of over 18% (Detailed photovoltaic parameters are summarized in Table S3, Supporting Information). c) EQE spectra of the binary and ternary OSCs. d–f) The sEQE and EL spectra of the binary and ternary OSCs. g) Orbital energy diagram of PM6:D18‐Cl:L8‐BO ternary system with alloying donor. h) Illustration of the potential energy surfaces for ground state (GS), LE state and CT state. $k_{\mathrm{nr}}^{\mathrm{CT}-\mathrm{LE}}\;\mathrm{and}\;k_{\mathrm{nr}}^{\mathrm{LE}-\mathrm{CT}}$ are the nonradiative transitions from CT/LE state to LE/CT state. $k_{r}^{\mathrm{LE}}$, $k_{r}^{\mathrm{CT}}$, or $k_{\mathrm{nr}}^{\mathrm{LE}}$, $k_{\mathrm{nr}}^{\mathrm{CT}}$ is the radiative or nonradiative transitions from the LE/CT state to the ground state. i) EQE_EL_ curves of the binary and optimal ternary OSCs.

Based on the good miscibility of PM6 and D18‐Cl, the binary and ternary OSCs were constructed with a conventional configuration of ITO/PEDOT:PSS/active layer/PDIN/Ag (Figure S3, Supporting Information). The donor/acceptor (D/A) weight ratio was kept constant (1:1.2), and the detailed experimental procedure is described in the Supporting Information. The current density–voltage (*J*–*V*) curves of all OSCs under standard AM 1.5G illumination with 100 mW cm^−2^ were shown in **Figure** **2**a; and Figure S4 (Supporting Information), and the detailed photovoltaic parameters were summarized in **Table** **1**; and Table S2 (Supporting Information). The PM6‐based binary OSCs delivered a PCE of 18.29%, with a *J*_sc_ of 26.35 mA cm^−2^, a *V*_oc_ of 0.885 V, and an FF of 78.45%, while D18‐Cl‐based ones achieved a PCE of 17.12%, with a high *V*_oc_ of 0.940 V and relatively low *J*_sc_ and FF of 25.38 mA cm^−2^ and 72.16%, respectively. Obviously, D18‐Cl‐based binary OSCs exhibited low *E*_loss_ due to its high *V*_oc_, in comparison to the PM6‐based ones. Thus, efficient ternary OSCs with low *E*_loss_ and high *V*_oc_ can be expected by employing alloyed PM6:D18‐Cl as a donor, and L8‐BO as an acceptor.

**Table 1:** Photovoltaic parameters and detailed energy losses of binary and ternary OSCs

Active layer^a)^	*J*_sc_ [mA cm^−2^]	*Cal. J*_sc_ [mA cm^−2^]	*V*_oc_ [V]	FF [%]	PCE [%]
PM6:L8‐BO	26.35 (26.31±0.28)	25.32	0.885 (0.885±0.003)	78.45 (78.43±0.32)	18.29 (18.21±0.09)
PM6:D18‐Cl:L8‐BO	26.66 (26.59±0.22)	25.61	0.910 (0.907±0.004)	79.24 (78.86±0.37)	19.22 (19.08±0.12)
D18‐Cl:L8‐BO	25.38 (25.32±0.18)	24.50	0.940 (0.935±0.002)	72.16 (71.86±0.40)	17.12 (17.01±0.13)

^a)^ Average values with standard deviation were obtained from ten individual cells.

A series of ternary OSCs were fabricated with different contents of D18‐Cl in active layers. Apparently, the *V*_oc_ of ternary OSCs continuously improved with the increase of D18‐Cl content, which is a common characteristic observation in ternary OSCs with an alloy‐like model,^[^ ^41^, ^42^ ^]^ while the *J*_sc_ and FF increased first and then decreased gradually. When the weight ratio of PM6:D18‐Cl:L8‐BO is 0.7:0.3:1.2, the ternary OSCs display a highest PCE of 19.22%, with a *J*_sc_ of 26.66 mA cm^−2^, a *V*_oc_ of 0.910 V, and an FF of 79.24%. The *V*_oc_ of 0.910 V is the highest value for the OSCs with over 19% efficiency as exhibited in Figure 2b, and the certified PCE is 18.8% from the National Institute of Metrology (NIM) in China (Figure S5, Supporting Information). The simultaneously enhanced *J*_sc_ and FF of the ternary OSCs should be mainly attributed to the optimized active layers morphology for efficient exciton dissociation and charge transport, which can be verified by the increased external quantum efficiency (EQE) values over the whole wavelength range in comparison to the binary ones (Figure 2c; and Figure S4, Supporting Information). The increased *V*_oc_ of ternary OSCs may be ascribed to reduced *E*_loss_ by incorporating D18‐Cl in active layers.

The detailed analysis was performed to explore the effect of D18‐Cl incorporation on the three parts of *E*_loss_ (Δ*E*_CT_, Δ*E*_rad_, Δ*E*_nonrad_),^[^ ^43^, ^44^ ^]^ and the corresponding values were summarized in **Table** **2**. The *E*_g_ is determined to be 1.455, 1.456, and 1.459 eV for the PM6‐based, PM6:D18‐Cl:L8‐BO (0.7:0.3:1.2), and D18‐Cl‐based blends, respectively, according to the crossing point between the normalized absorption and emission spectra of the blend films (Figure S6, Supporting Information). The Δ*E*_rad_ values of binary and ternary OSCs are similar, which is inevitable and less tunable. The *E*_CT_ of the binary and ternary OSCs can be obtained by fitting highly sensitive EQE (sEQE) and electroluminescence (EL) spectra (Figure 2d–f). The *E*_CT_ values are elevated from 1.375 eV for PM6‐based binary OSCs to 1.387 eV for the ternary OSCs, and the corresponding Δ*E*_CT_ values are reduced from 0.080 to 0.069 eV. The elevated *E*_CT_ and thus reduced Δ*E*_CT_ of the ternary OSCs may be ascribed to the lower‐lying HOMO energy level of PM6:D18‐Cl alloy donor in active layers, resulting in the reduced HOMO–HOMO offset between donor and acceptor (Δ*E*_HOMO(D–A)_) (Figure 2g). In addition, the reduced Δ*E*_CT_ of ternary OSCs can facilitate the hybridizations between the LE state and CT state as exhibited in Figure 2h, which can increase the luminescence of CT state by borrowing the highly luminescent LE state.^[^ ^45^ ^]^ Also, the small Δ*E*_CT_ of ternary OSCs facilitates the transition from the CT state back to the LE state, which enables the CT state to undergo radiative recombination via the emissive LE state.^[^ ^46^ ^]^ Thus, the enhanced CT state luminescence of ternary OSCs can effectively reduce the Δ*E*_nonrad_. To confirm this mechanism, the EQE_EL_ of binary and ternary OSCs was measured to investigate the Δ*E*_nonrad_, which can be calculated according to the equation: Δ*E*_nonrad_ = −*kT*ln (EQE_EL_). As shown in Figure 2i, the PM6‐based binary OSCs exhibits an EQE_EL_ of 1.14 × 10^−4^, corresponding to Δ*E*_nonrad_ of 0.227 eV. While the ternary OSCs shows a higher EQE_EL_ of 2.44 × 10^−4^, leading to a lower Δ*E*_nonrad_ of 0.208 V. Based on the above analysis, the significantly improved *V*_oc_ of the ternary OSCs can be attributed to the elevated *E*_CT_ by the incorporation of D18‐Cl, resulting in the simultaneously reduced Δ*E*_CT_ and Δ*E*_nonrad_.

**Table 2:** Total and detailed energy losses of binary and ternary OSCs

Active layer	*E*_g_ [eV]	*E*_loss_ [eV]	Δ*E*_rad_ [eV]	*E*_CT_ [eV]	Δ*E*_CT_ [eV]	EQE_EL_	Δ*E*_nonrad_ [eV]
PM6:L8‐BO	1.455	0.57	0.263	1.375	0.080	1.14E‐4	0.227
PM6:D18‐Cl:L8‐BO	1.456	0.546	0.269	1.387	0.069	2.44E‐4	0.208
D18‐Cl:L8‐BO	1.459	0.519	0.265	1.394	0.065	5.24E‐4	0.189

**Figure 3:**
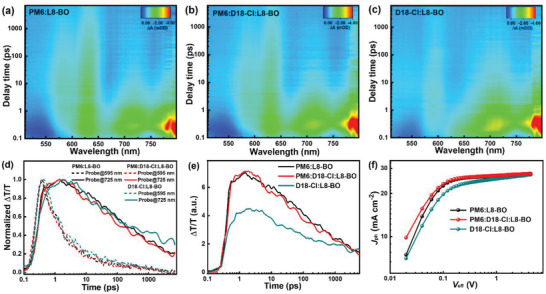
a–c) 2D TA color plots of binary and ternary blend films. d) The decay traces of donor and acceptor in binary and ternary blend films, monitored at 595 and 725 nm, respectively. e) The GSB kinetics of donor probed at 595 nm in binary and ternary blend films. f) *J*_ph_–*V*_eff_ curves of the binary and ternary OSCs.

The small Δ*E*_CT_ in the OSCs signifies that the driving force for exciton dissociation within the device may be low. To investigate the effect of the reduced Δ*E*_CT_ on exciton dissociation processes in the ternary OSCs, transient absorption (TA) spectroscopy measurements were performed to probe the photo‐induced hole transfer dynamics in binary and ternary blend films. Figure S7 (Supporting Information) shows the TA spectra of neat L8‐BO film, where the negative signals in the range of 650–790 nm are assigned to its ground‐state bleach (GSB). **Figure** **3**a–c exhibits the 2D TA color plots of binary and ternary blend films. With the decay of L8‐BO GSB signal in the range of 650–790 nm, the donors GSB signal at 560–650 nm emerges in the binary and ternary films. The signals at 595 and 725 nm were selected to investigate the decay traces of donor and acceptor, respectively. As shown in Figure 3d, the decay of the L8‐BO GSB signal at 725 nm agrees with the rise of the GSB signal of the donor at 595 nm, implying the photoinduced hole transfer process from L8‐BO to the donor.^[^ ^47^, ^48^ ^]^ It is obviously observed that the GSB signals at 595 nm for the PM6‐based and the ternary films took the same time to reach saturation values, indicating the similar exciton dissociation rates within these two films.^[^ ^49^ ^]^ Thus, the reduced Δ*E*_CT_ value in the ternary OSCs exhibited no effect on the exciton dissociation processes, suggesting that ternary alloy strategy is an efficient way of delicately tuning the *E*_CT_ of OSCs, while maintaining sufficient driving force for exciton dissociation. The GSB signal intensity at 595 nm of the blend films should be positively correlated with the amount of hole transfer from L8‐BO to the donor, which can reflect the exciton dissociation efficiency within the blend film. As shown in Figure 3e, the PM6‐based blend film and the optimized ternary films exhibit the relatively strong GSB signals at 595 nm, while a relatively weak GSB signal was observed in D18‐Cl‐based blend film, indicating the inferior exciton dissociation efficiency in the D18‐Cl‐based blend film.

To further investigate the exciton dissociation properties in these films, the photocurrent density (*J*_ph_) of the OSCs were measured under different effective bias (*V*_eff_) as shown in Figure 3f. The exciton dissociation and charge collection efficiency can be reflected by *J*_ph_*/*J*_sat_ and *J*_ph_^#^/*J*_sat_, respectively. The *J*_ph_* and *J*_ph_^#^ are the *J*_ph_ under short‐circuit and maximum power‐output conditions, respectively, while *J*_sat_ represents the *J*_ph_ under the condition that all photogenerated excitons are dissociated into free charge and then collected by the individual electrodes.^[^ ^50^, ^51^ ^]^ The detailed values of the typical OSCs are summarized in Table S4 (Supporting Information). The relatively large *J*_sat_ of ternary OSCs should be attributed to the slightly broadens photon harvesting range of active layer by incorporating D18‐Cl. The exciton dissociation efficiencies and the charge collection efficiencies are 96.73%/89.64%, 93.65%/82.14%, and 96.94%/90.47% for the PM6‐, D18‐Cl‐based binary OSCs, and the ternary OSCs, respectively. The simultaneous enhancement of exciton dissociation and charge collection efficiency in the ternary device can well explain its improved *J*_sc_ and FF.

**Figure 4:**
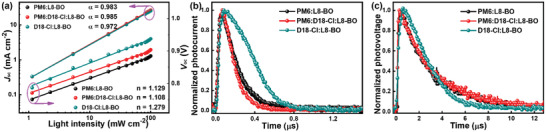
a) *V*_OC_ and *J*_SC_ of the OSCs dependence on *P*_light_. b) TPC curves and c) TPV curves of the binary and ternary OSCs.

To investigate the charge recombination in binary and ternary OSCs, the *V*_oc_ and *J*_sc_ dependence on light intensity (*P*_light_) were measured, and the corresponding characteristics are plotted in **Figure** **4**a. The dependence of *J*_sc_ on *P*_light_ can be expressed as *J*_sc_ ∝ *P*_light_ *^*α*^*, while the bimolecular recombination can be completely suppressed when *α* value is equal to 1. The relationship of *V*_oc_ dependence on *P*_light_ can be described as *V*_oc_ ∝ *n*(*kT/q*)ln*P*_light_, whereas the trap‐assisted recombination can be negligible if *n* value is close to 1.^[^ ^52^, ^53^ ^]^ The *α* and *n* values are 0.983/1.129, 0.972/1.279, and 0.986/1.108 for the PM6‐, D18‐Cl‐based binary OSCs, and the optimal ternary OSCs, respectively. Both the *α* and *n* values of the ternary OSCs are closer to 1 than those of binary devices, indicating the bimolecular and trap‐assisted recombination within the device can be weakened. The space‐charge limited current (SCLC) method was employed to investigate the charge transport mobilities of binary and ternary blend films. The ln(*Jd^3^/V^2^*)−(*V/d*)^0.5^ curves of hole‐only and electron‐only devices are depicted in Figure S8 (Supporting Information), and the calculated hole mobility (*µ*_h_) and electron mobility (*µ*_e_) are listed in Table S5 (Supporting Information). Compared with the two binary blend films, both *µ*_h_ and *µ*_e_ in the ternary blend films were improved, resulting in more balanced charge transport mobilities (*µ*_h_/*µ*_e_ = 1.02). To gain more insights into the charge transport dynamic processes in active layers, transient photocurrent (TPC) and transient photovoltage (TPV) measurements were performed (Figure 4b,c). The charge extraction time derived by fitting the TPC curves were 0.16, 0.21, and 0.12 µs for the PM6‐, D18‐Cl‐based binary OSCs, and the ternary OSCs, while the carrier lifetimes extracted from the TPV decay dynamics were 2.82, 2.11, and 2.94 µs for the corresponding OSCs, respectively. The shorter charge extraction time and longer carrier lifetime of the ternary OSCs signify higher charge mobility and less charge recombination in the active layers, which is beneficial to improve the *J*_sc_ and FF of OSCs.^[^ ^54^, ^55^ ^]^

Grazing‐incidence wide‐angle X‐ray scattering (GIWAXS) was performed to explore the effect of D18‐Cl on intermolecular packing in ternary blend films. Figure S9 (Supporting Information) displays the 2D GIWAXS patterns and 1D line‐cut profiles of neat PM6, D18‐Cl, and L8‐BO films. The neat L8‐BO film exhibits an obvious *π*–*π* stacking peak at 1.73 Å^−1^ in the out‐of‐plane (OOP) direction and lamellar stacking peaks at 0.38 and 0.45 Å^−1^ in the in‐plane (IP) direction, suggesting a preferred face‐on orientation of L8‐BO.^[^ ^56^, ^57^ ^]^ A dominant lamellar stacking peak at *q* ≈ 0.30 Å^−1^ in OOP direction and a relatively weak lamellar stacking peak at 0.29 Å^−1^ in IP direction were observed in the GIWAXS files of neat PM6 films, indicating the coexistence of face‐on and edge‐on orientation of PM6, similar to the reported results.^[^ ^31^ ^]^ Moreover, the neat D18‐Cl film adopts face‐on orientation with an obvious *π*–*π* stacking peak at 1.65 Å^−1^ in OOP direction and a lamellar stacking peak at 0.31 Å^−1^ in IP direction.

**Figure 5:**
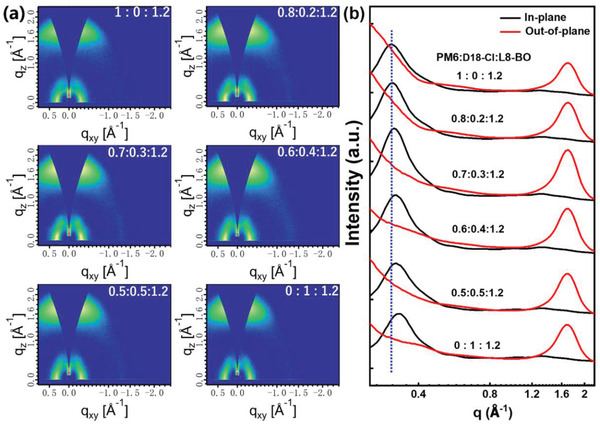
a) 2D GIWAXS patterns and b) 1D line‐cut profiles of PM6:D18‐Cl:L8‐BO films with various D18‐Cl contents.

**Figure** **5**a,b displays the 2D‐GIWAXS patterns and 1D line‐cut profiles of the blend films. All the blend films exhibit a preferential face‐on orientation with distinct lamellar stacking peaks in IP direction and *π*–*π* stacking peak in OOP direction, which is beneficial to charge transport in OSCs. According to the diffraction vector (*q*) values, the IP direction lamellar stacking peaks at 0.31 Å^−1^ in PM6‐based blend films should be associated with PM6, while the IP direction lamellar stacking peaks at 0.33 Å^−1^ in D18‐Cl‐based blend films is associated with D18‐Cl. In the ternary blend films, the IP direction lamellar stacking peaks associated with PM6 gradually shift to larger *q* values with the increase of the amount of D18‐Cl, revealing that PM6 and D18‐Cl can form a well‐mixed crystalline phase in ternary blend films. In addition, compared with the two binary blend films, the lamellar stacking peaks and *π*–*π* stacking peak in the optimal ternary blend films were simultaneously enhanced. Meanwhile, the crystal coherence lengths (CCLs, calculated via Scherrer equation) of the corresponding lamellar stacking and *π*–*π* stacking peaks in ternary blend films are larger than that of the two binary blend films, suggesting the enhanced intermolecular stacking in the former system. This feature may facilitate the charge transport in ternary blend films, which is well consistent with the increased *J*_sc_ and FF of the corresponding OSCs. Transmission electron microscopy (TEM) was carried out to further investigate the influence of D18‐Cl incorporation on phase separation in ternary blend films. As shown in Figure S10 (Supporting Information), the slender phase separation is uniformly distributed in the PM6‐based blend film, while the excessive phase separation can be observed from the TEM image of the D18:L8‐BO film. The phase separation degree of the ternary blend films can be gradually enlarged with increasing the D18‐Cl content, indicating that D18‐Cl can act as a morphology regulator to finely tune the phase separation in ternary blend films.

## 3 Conclusion

In summary, high‐performance ternary OSCs were prepared with PM6:D18‐Cl as the alloyed donor and L8‐BO as the acceptor. The formation of PM6:D18‐Cl donor alloy enabled precisely tuning the *E*_CT_ by simply varying its composition ratio, resulting in reduced Δ*E*_CT_ and Δ*E*_nonrad_, and significantly enhanced *V*_oc_ of the ternary OSCs. Simultaneously, the broadened optical absorption range and well optimized molecular packing and phase separation in the ternary films efficiently improved the *J*_sc_ and FF. As a result, the ternary OSCs delivered a much higher PCE of 19.22% with increased *J*_sc_ of 26.66 mA cm^−2^, *V*_oc_ of 0.910 V and FF of 79.24% in comparison to that of the PM6‐based binary OSCs. This contribution presented that precisely tuning the *E*_CT_ via donor alloy strategy is an efficient strategy to achieve highly efficient OSCs.

## Conflict of Interest

The authors declare no conflict of interest.

## Supporting information

Supporting Information

## Data Availability

The data that support the findings of this study are available from the corresponding author upon reasonable request.
