# Supplementary material for: Over 19.2% Efficiency of Organic Solar Cells Enabled by Precisely Tuning the Charge Transfer State Via Donor Alloy Strategy
Source: Adv Sci (Weinh). 2022 Aug 23;9(30):2203606. doi: 10.1002/advs.202203606 (PMC9596821; doi:10.1002/advs.202203606)
Supplement: Supplementary file 1 — Supporting Information [file ADVS-9-2203606-s001.pdf]

Over 19.2% efficiency of organic solar cells enabled by precisely tuning the charge transfer state via donor alloy strategy

Jinhua Gao, Na Yu, Zhihao Chen, Yanan Wei, Congqi Li, Tianhua Liu, Xiaobin Gu, Jianqi Zhang, Zhixiang Wei, Zheng Tang, Xiaotao Hao, Fujun Zhang, Xin Zhang, and Hui Huang**

J. Gao, Y. Wei, C. Li, T. Liu, X. Gu, Prof. X. Zhang, Prof. H. Huang
College of Materials Science and Opto-Electronic Technology
Center of Materials Science and Optoelectronics Engineering
CAS Center for Excellence in Topological Quantum Computation
CAS Key Laboratory of Vacuum Physic
University of Chinese Academy of Sciences
Beijing 100049, China
E-mail: huihuang@ucas.ac.cn, zhangxin2019@ucas.ac.cn

N. Yu, Prof. Z. Tang
Center for Advanced Low-Dimension Materials
State Key Laboratory for Modification of Chemical Fibers and Polymer Materials
College of Materials Science and Engineering
Donghua University
Shanghai 201620, China

Z. Chen, Prof. X. Hao
School of Physics
State Key Laboratory of Crystal Materials
Shandong University
Jinan, Shandong 250100, China

Prof. J. Zhang, Prof. Z. Wei
Center for Excellence in Nanoscience (CAS)
Key Laboratory of Nanosystem and Hierarchical Fabrication (CAS)
National Center for Nanoscience and Technology
Beijing 100190, China

Prof. F. Zhang
Key Laboratory of Luminescence and Optical Information
Ministry of Education, Beijing Jiaotong University
Beijing,100044, PR China

1. Experimental Section

Materials:

The PDIN, PM6, and D18-Cl were purchased from Solarmer Materials Inc., L8-BO was provided by Derthon. Poly(3,4-ethylenedioxythiophene):poly(styrene sulfonate) (PEDOT:PSS, clevios PVP Al 4083) was purchased from H.C. Starck Co. Ltd and used as received.

Sample characterization:

The UV-vis absorption spectra were obtained on an Agilent Cary 60 UV-vis Spectrophotometer. GIWAXS measurements were carried out with a Xeuss 2.0 SAXS/WAXS laboratory beamline using an in-door Cu X-ray source (8.05 keV) and a Pilatus3R 300K detector. Transmission electron microscopy (TEM) images of active layers were obtained by a JEOL JEM-1400 transmission electron microscope operated at 80 kV.

The contact angle measurements were performed by Rame-Hart goiniometer in sessile drop mode. The contact angles of neat PM6, D18-Cl and L8-BO films were measured based on water and ethylene glycol. The surface energy of neat films were calculated according to the contact angles by using Wu model. According to the surface energy of neat films, the interfacial energy between two different materials in the blend films can be evaluated by the following equation: [1, 2]

$$\gamma_{\mathrm{X/Y}} = \gamma_{\mathrm{XP}} + \gamma_{Y} - \frac{4\gamma_{\mathrm{X}}^{\mathrm{d}}\gamma_{\mathrm{Y}}^{\mathrm{d}}}{\gamma_{\mathrm{X}}^{\mathrm{d}} + \gamma_{\mathrm{Y}}^{\mathrm{d}}} - \frac{4\gamma_{\mathrm{X}}^{\mathrm{p}}\gamma_{\mathrm{Y}}^{\mathrm{p}}}{\gamma_{\mathrm{X}}^{\mathrm{p}} + \gamma_{\mathrm{Y}}^{\mathrm{p}}}$$

Here, $\gamma_{\mathrm{X/Y}}$ is the interfacial energy between material X and Y; γ_{X} and γ_{Y} are the surface tension of X and Y, and superscript d and p represent the dispersion and polar components calculated by using the contact angles.

Cyclic voltammetry (CV) measurement was carried out on a CHI voltammetric analyzer at room temperature. Tetrabuty-lammonium hexafluorophosphate (*n*-Bu_4NPF_6, 0.1 M) was used as the supporting electrolyte. The conventional three-electrode configuration consists of a platinum working electrode with a 2 mm diameter, a platinum wire counter electrode, and an Ag/AgCl wire reference electrode.

CV curves were obtained at a scan rate of 100 mV s^{-1}. The potentials were determined using ferrocene as the reference. The HOMO and LUMO energy levels were calculated according to the following equations:

$$E_{\mathrm{HOMO}} = -[E_{\mathrm{ox}} + (4.80 - E_{\mathrm{Fc}})]\mathrm{eV}$$

$$E_{\mathrm{LUMO}} = -[E_{\mathrm{red}} + (4.80 - E_{\mathrm{Fc}})]\mathrm{eV}$$

where E_{ox} and E_{red} are the onset of oxidation and reduction potential, respectively.

TA measurements were performed on an Ultrafast Helios pump-probe system in collaboration with a regenerative amplified laser system from Coherent. An 800 nm pulse with a repetition rate of 1k Hz, a length of 100 fs, and an energy of 7 mJ pluse-1, was generated by an Ti:sapphire amplifier (Astrella, Coherent). Then the 800 nm pulse was separated into two parts by a beam splitter. One part was coupled into an optical parametric amplifier (TOPAS, Coherent) to generate the pump pulses at various wavelengths. The other part was focused onto a sapphire plate and a YAG plate to generate white light supercontinuum as the probe beams with spectra covering 420-800 nm and 750-1600 nm, respectively. The time delay between pump and probe was controlled by a motorized optical delay line with a maximum delay time of 8 ns. The pump pulse is chopped by a mechanical chopper with 500 Hz and then focused on to the mounted sample with probe beams. The probe beam was collimated and focused into a fiber-coupled multichannel spectrometer with CCD sensor. The energy of pump pulse was measured and calibrated by a power meter (PM400, Thorlabs). The samples used for TA measurements were obtained by spin-coating the neat and blend solutions on the quartz substrates.

Device Fabrication and characterization:

Organic solar cells (OSCs) were fabricated with a structure ITO/PEDOT:PSS/active layers/PDIN/Ag. The patterned indium tin oxide (ITO) glass coated substrates (sheet resistance 15 Ω/□) were pre-cleaned by sequential ultrasonic treatment in detergent, deionized water and ethanol, respectively. The cleaned ITO substrates were blow-dried by high pure nitrogen gas and then treated by oxygen

plasma for 1 min. Subsequently, PEDOT:PSS solution was spin-coated on the ITO substrates at 5000 round per minute (RPM) for 30 s and then annealed at 150 ℃ for 15 min in air. After annealing, the PEDOT:PSS-coated ITO substrates were transferred to a high-purity nitrogen-filled glove box to fabricate active layer. The PM6, D18-Cl and L8-BO were dissolved in chloroform to prepare 17 mg/ml blend solutions, and 0.5 vol% diiodomethane was added as the additive. The weight ratios of PM6:D18-Cl:L8-BO are 1:0:1.2, 0.8:0.2:1.2, 0.7:0.3:1.2, 0.6:0.4:1.2, 0.5:0.5:1.2, and 0:1:1.2. After heated and stirred at 45℃ for 3 h, the blend solutions were spin-coated on PEDOT:PSS films. Next, the active layers were upside-down solvent vapor annealed with carbon disulfide for 30 s and then thermal annealed at 80 °C for 5 min. Afterwards, PDIN solution (2 mg/ml in methanol with 0.25 vol% acetic acid) was spin-coated on top of the active layers at 5000 rpm for 30 s to prepare cathode interlayer. Finally, Ag (100 nm) was deposited by thermal evaporation with a shadow mask under 10^{-4} Pa, monitored by a quartz crystal microbalance. The active area is approximately 4 mm^2, which is defined by the overlapping area of ITO anode and Ag cathode.

The cells were characterized under a temperature of 25–30 °C in a glove box filled with nitrogen. The current density–voltage (*J*–*V*) characteristics of photovoltaic devices were obtained along the forward scan direction from -0.5 to 1 V, with a scan step of 0.02 V/s and a dwell time of 1 ms using a Keithley 2400 source-measure unit. The photocurrent was measured under illumination simulated 100 mW cm^{-2} AM1.5G irradiation using a Newport solar simulator. Simulator irradiance was characterized using a calibrated spectrometer and illumination intensity was set using a certified silicon diode (SRC-2020, Enlitech). The area of the mask is 3.169 mm^2 in NIM, China, determined by an optical microscope. External quantum efficiency (EQE) values of the devices were measured using a QE-R3011 instrument (Enli Technology Co. Ltd., Taiwan) with a scan increment of 5 nm per point.

Transient photovoltage (TPV) and transient photocurrent (TPC) measurements are as follows: A white light bias was generated from an array of diodes (Molex

180081-4320) with light intensity about 0.5 sun. A diode pumped laser (Lapa-80) was used as the perturbation source, with a pulse duration of 10 ns and a repetition frequency of 20 Hz. The perturbation light intensity was attenuated to keep the amplitude of transient V_{oc} (ΔV_{oc}) below 10 mV so that $\Delta V_{oc} << V_{oc}$. Voltage and current dynamics were recorded on a digital oscilloscope (Tektronix MDO4104C), and voltages at open circuit and currents under short circuit conditions were measured over a 1 MΩ and a 50 Ω resistor, respectively.

Energy loss (E_{loss}) analysis: The E_{loss} of OSCs is defined as the difference between the bandgap (E_g) of the photoactive materials and the V_{OC} of the device, which can be classified into three parts: $E_{loss} = (E_g - E_{CT}) + \Delta E_{rad} + \Delta E_{non-rad} = \Delta E_{CT} + \Delta E_{rad} + \Delta E_{non-rad}$. The E_g is determined by the crossing point between the normalized absorption and emission spectra of the blend films.

Sensitive EQE (sEQE) measurement was performed as follows: A 150 W quartz halogen lamp (LSH-75, Newport) acted as a light source, passing through the monochromator (CS260-RG-3-MC-A, Newport) to provide an adjustable monochromatic light source for testing, and then emited an optical signal at a 173 Hz frequency through the chopper (3502 Optical Chopper, Newport) and focused on the OSC devices. The current generated by the device was amplified by the front-end current amplifier (SR570, Stanford) to reduce the impact of the noise signal. The final signal was collected and analysed by a Phase-locked Amplifier (SR830 DSP Lock-In Amplifier, Stanford).

Fitting of E_{CT}: The tails of the EQE spectra are determined by a sensitive EQE setup, which consists a halogen lamp, a monochromator, a current amplifier and a lock-in amplifier. A set of long pass filters are used to cut the higher order wavelengths from the monochromator, which generated monochromatic light for test. To further extend the range of the EQE spectra measured by the sensitive EQE setup, electroluminescence (EL) spectra of the solar cells are also measured, using a small injection current. Since EL spectra of the OSCs are dominated by CT state emission, they can be converted to CT state absorption spectra using the reciprocal relation. The

tail of the EQE spectrum, corresponding to CT absorption, was used to determine the energy of CT state (E_{CT}) by fitting the equation derived in the framework of Marcus theory.

$$EL(E) = EQE(E)\phi_{BB}(E)[\exp\left(\frac{qV}{kT}\right) - 1] \qquad (2)$$

$$EQE(E) = \frac{fE}{\sqrt{4\pi\lambda kT}}\exp(-\frac{(E_{CT}+\lambda+E)^2}{4\lambda kT}) \qquad (3)$$

To avoid an arbitrary fitting, two boundary conditions are imposed. First, we calculate the lower limit for the radiative recombination voltage loss ($V_{r,sq}$) for a solar cell as a function of E_{CT} using the Shockley-Quessior theory, assuming that E_{CT} is the effective energy of the bandgap of an OSCs. Then, the Gaussian region in the lower energy part of the EQE spectrum is selected and fitted. During the fitting process, a set of f values typically in the range between 0.0001 to 0.1 are used as constant input values, while E_{CT} and λ are left as fit parameters.

The space charge limited current (SCLC) method was employed to investigate the charge mobility of binary and ternary OSCs. The structure of electron-only devices is ITO/ZnO/active layer/PDIN/Al and the structure of hole-only devices is ITO/PEDOT:PSS/active layer/MoO_3/Ag. The fabrication conditions of the active layer films are same with those for the solar cells. The charge mobility was calculated according to the SCLC method. The hole and electron mobility can be calculated from the Mott-Gurney equation with Poole-Frenkel correction as the followings:

$$J = \frac{9}{8}\varepsilon_0\varepsilon_r\mu\frac{V^2}{d^3}exp\left[0.89\gamma\sqrt{\frac{V}{d}}\right]$$

Here, ε_r is dielectric constant of organic materials, ε_0 is the free space permittivity, μ is charge mobility, V is the applied voltage, and d is the active layer thickness, γ is the field enhancement factor of a Poole–Frenkel type mobility.

2. Supporting Figures

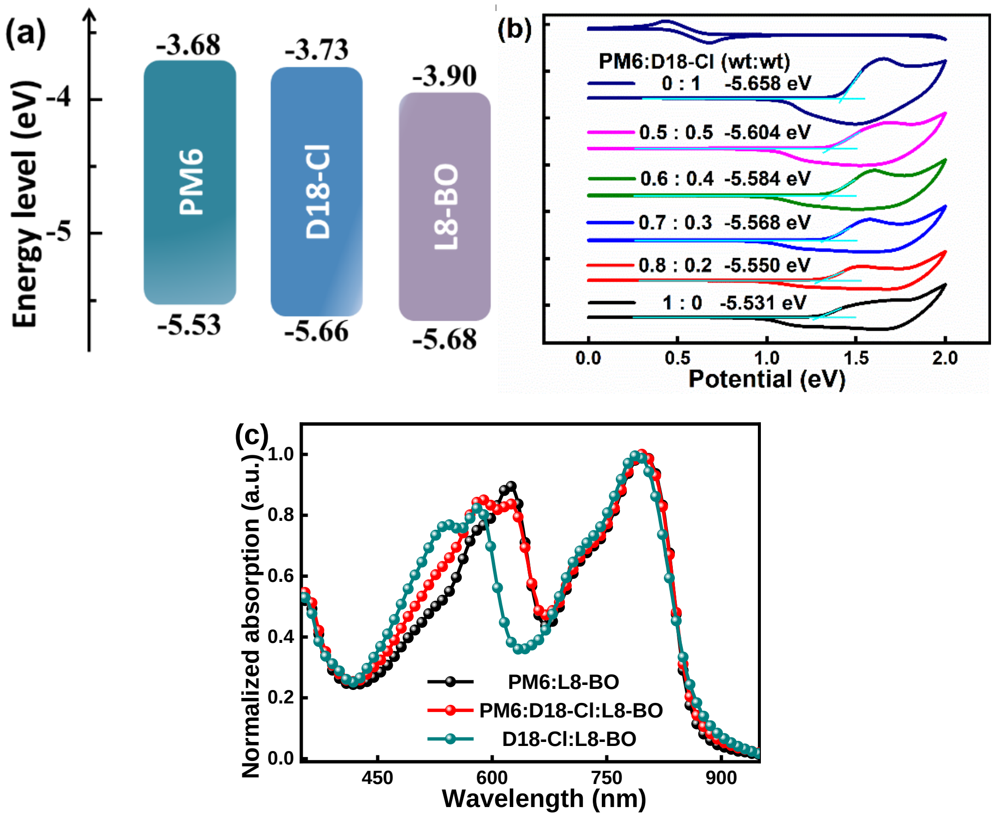

Figure S1 (a) Energy levels of PM6, D18-Cl, and L8-BO; (b) Cyclic voltammetry of PM6:D18-Cl blend with different D18-Cl contents; (c) Normalized absorption spectra of PM6:L8-BO (1:1.2), PM6:D18-Cl:L8-BO (0.7:0.3:1.2) and D18-Cl:L8-BO (1:1.2) blend films.

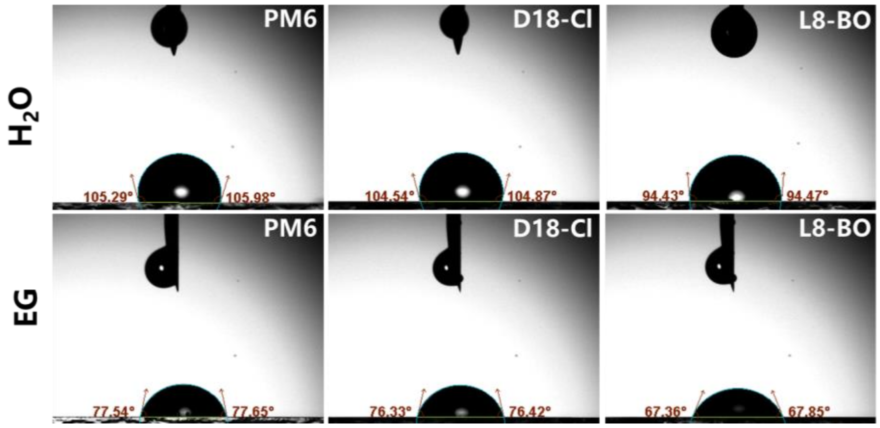

Figure S2 Contact angle images of neat PM6, D18-Cl and L8-BO films.

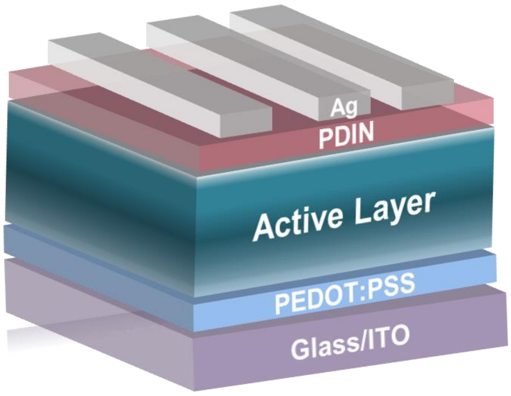

Figure S3 Schematic diagram of device structure.

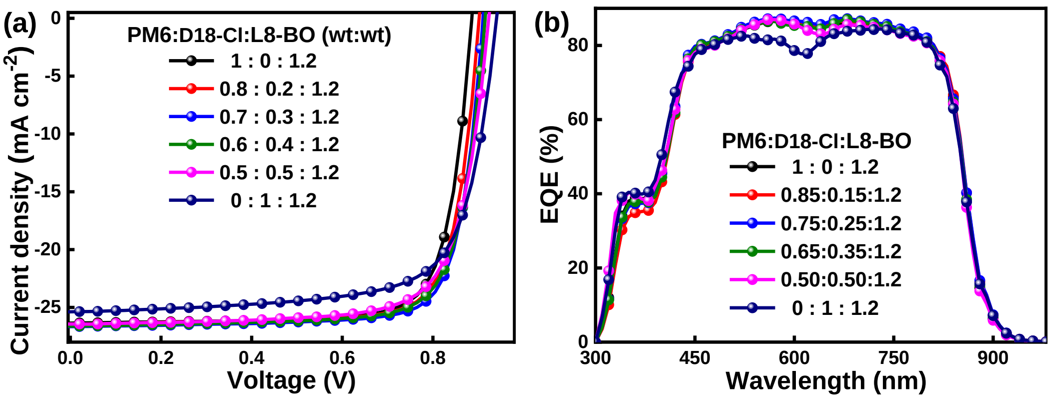

Figure S4 (a) *J-V* curves, (b) EQE spectra of PM6:D18-Cl:L8-BO based OSCs with different D18-Cl contents.

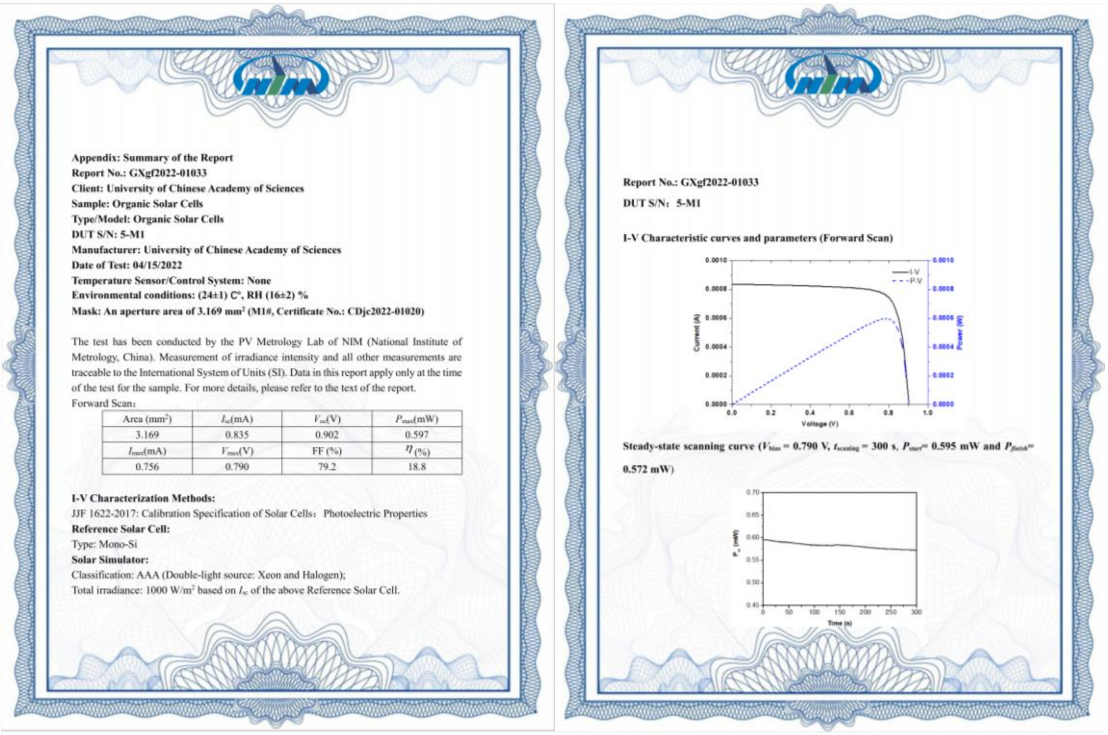

Appendix: Summary of the Report
Report No.: GXgf2022-01033
Client: University of Chinese Academy of Sciences
Sample: Organic Solar Cells
Type/Model: Organic Solar Cells
DUT S/N: 5-M1
Manufacturer: University of Chinese Academy of Sciences
Date of Test: 04/15/2022
Temperature Sensor/Control System: None
Environmental conditions: (24±1) C°, RH (16±2) %
Mask: An aperture area of 3.169 mm^2 (M1#, Certificate No.: CDjc2022-01020)

The test has been conducted by the PV Metrology Lab of NIM (National Institute of Metrology, China). Measurement of irradiance intensity and all other measurements are traceable to the International System of Units (SI). Data in this report apply only at the time of the test for the sample. For more details, please refer to the text of the report.

Forward Scan:

Area (mm^2)	I_{sc}(mA)	V_{oc}(V)	P_{max}(mW)
3.169	0.835	0.902	0.597
I_{max}(mA)	V_{max}(V)	FF (%)	η (%)
0.756	0.790	79.2	18.8

I-V Characterization Methods:
JJF 1622-2017: Calibration Specification of Solar Cells: Photoelectric Properties
Reference Solar Cell:
Type: Mono-Si
Solar Simulator:
Classification: AAA (Double-light source: Xeon and Halogen);
Total irradiance: 1000 W/m^2 based on I_{sc} of the above Reference Solar Cell.

Report No.: GXgf2022-01033
DUT S/N: 5-M1

I-V Characteristic curves and parameters (Forward Scan)

Steady-state scanning curve (V_{bias} = 0.790 V, $t_{scaning}$ = 300 s, P_{start} = 0.595 mW and P_{finish} = 0.572 mW)

Figure S5 NIM certification report of optimal ternary OSCs.

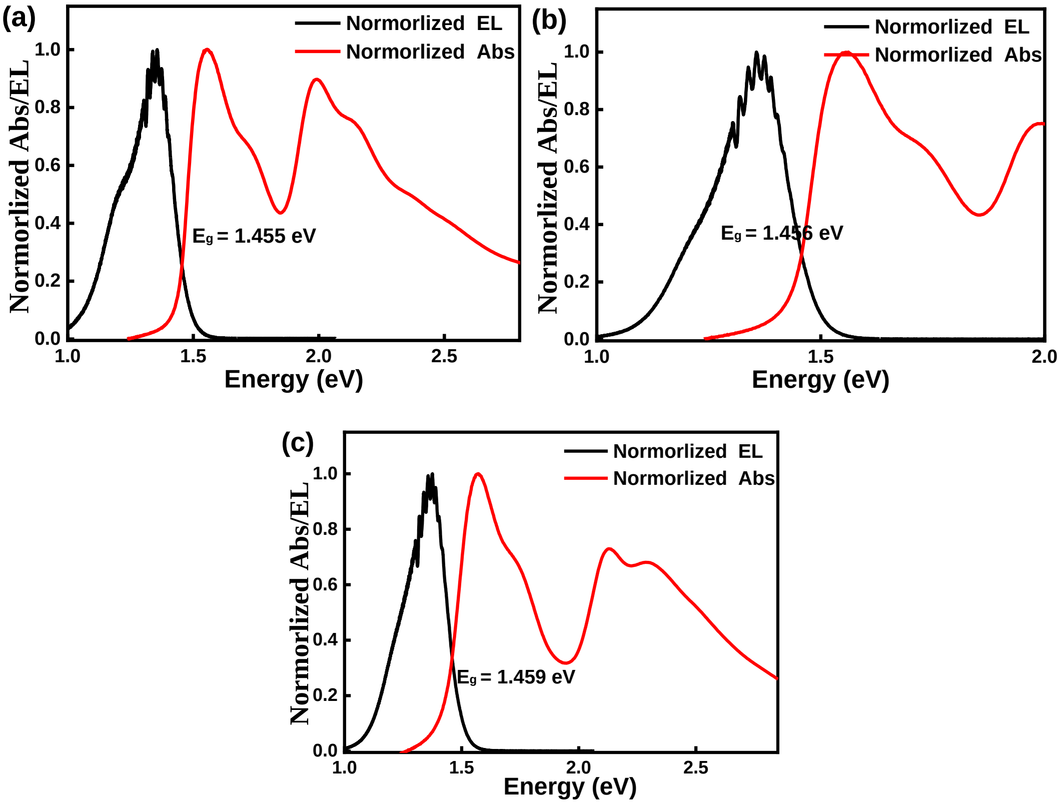

Figure S6 Determination of E_g by the intersection of UV-vis absorption and PL emission of blend films: (a) PM6:L8-BO, (b) PM6:D18-Cl:L8-BO, and (c) D18-

Cl:L8-BO.

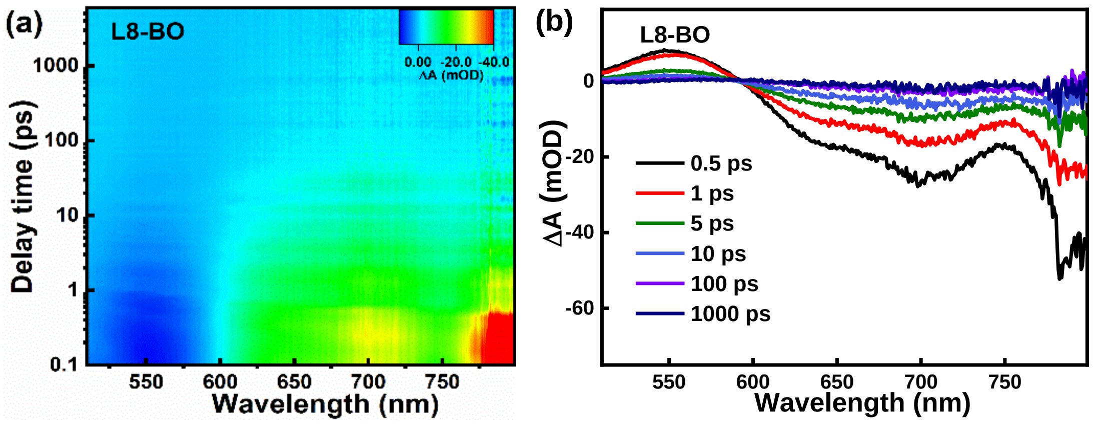

Figure S7 (a) 2D TA color plots of L8-BO films; (b) TA spectra with varied delay times of L8-BO films.

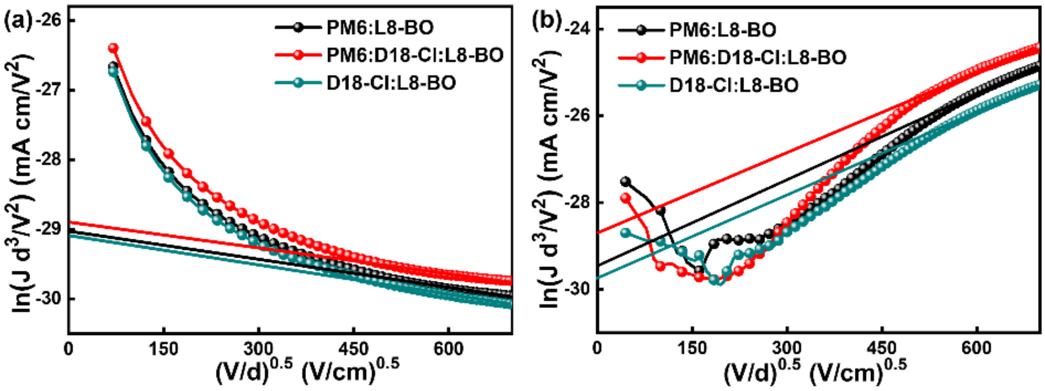

Figure S8. The ln(Jd^3/V^2) $(V/d)^{0.5}$ versus $(V/d)^{0.5}$ curves of (a) hole-only devices and (b) electron-only devices.

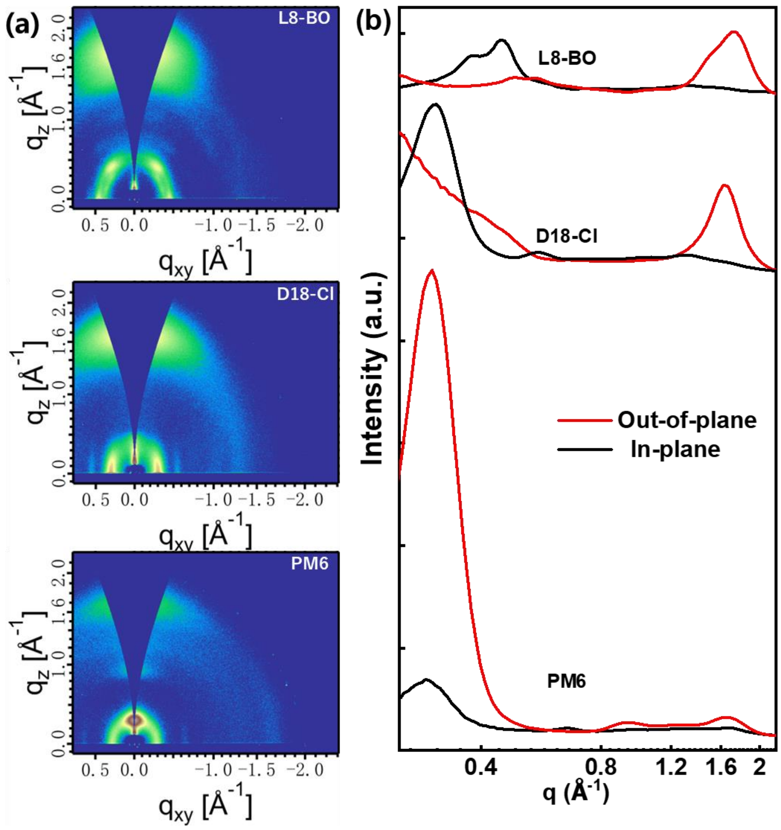

Figure S9 (a) 2D GIWAXS patterns and (b) 1D line-cut profiles of neat PM6, D18-Cl and L8-BO films.

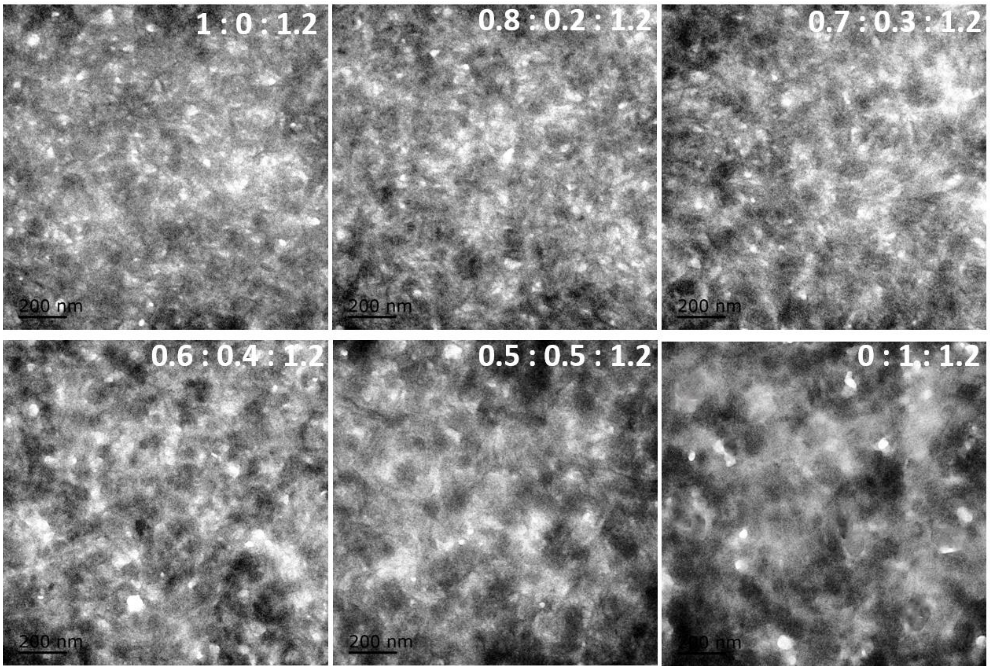

Figure S10 TEM images of the blend films with various D18-Cl content.

3. Supporting Tables

Table S1. Contact angle, surface tension (γ) of individual materials, and interfacial tension between two materials ($\gamma_{X/Y}$).

Film X	Contact angle H_2O (deg)	Contact angle EG (deg)	γ (mN m^{-1})	γ^d (mN m^{-1})	γ^p (mN m^{-1})	Film Y	$\gamma_{X/Y}$ (mN m^{-1})
L8-BO	94.45	67.61	25.47	17.07	2.37	PM6	3.51
D18-Cl	104.71	76.38	24.21	21.25	8.40	L8-BO	3.41
PM6	105.61	77.60	23.92	21.31	2.61	D18-Cl	0.02

Table S2. Photovoltaic parameters of PM6:D18-Cl:L8-BO based OSCs with different D18-Cl contents.

PM6:D18-Cl:L8-BO (wt:wt:wt)	J_{sc} (mA cm^{-2})	V_{oc} (V)	FF (%)	PCE (%)
1:0:1.2	26.35	0.885	78.45	18.29
0.8:0.2:1.2	26.43	0.902	78.55	18.72
0.7:0.3:1.2	26.66	0.910	79.24	19.22
0.6:0.4:1.2	26.62	0.916	77.32	18.85
0.5:0.5:1.2	26.43	0.921	74.78	18.20
0:1:1.2	25.38	0.940	72.16	17.22

Table S3. Summary of recently reported high-performance OSCs.

Active Layer	J_{sc} (mA cm^{-2})	V_{oc} (V)	FF (%)	PCE (%)	References
PM6:Y6:AQx-3	26.82	0.870	77.2	18.01	[1]
PM6:BPR-SCl:BTP-eC9	27.13	0.856	77.6	18.02	[2]
PM6:BTP-eC9:PC71BM	26.94	0.845	79.2	18.03	[3]
PM6:PM7:Y6:PC_{71}BM	26.55	0.859	79.23	18.07	[4]
PM6:Y6:PC_{71}BM:CNS-6-8	26.43	0.868	78.8	18.07	[5]
PM6-Ir1:Y6	27.01	0.862	76.13	18.07	[6]
PM6:Y6:ITIC-M	26.35	0.859	80.10	18.13	[7]
PM6:Y6:TIT-2Cl	26.63	0.876	77.93	18.18	[8]
PM6:BTP-4F-C5-16	27.78	0.844	77.68	18.20	[9]
D18:Y6	27.70	0.859	76.6	18.22	[10]
PBDB-T-2F: BTP-4F-P2EH	25.85	0.880	80.08	18.22	[11]
PM6:L8-BO	25.71	0.87	81.50	18.23	[12]

PM6:BTP-eC9:$PC_{71}BM$	26.93	0.856	79.4	18.3	[13]
PM6:L8-BO	25.72	0.87	81.5	18.32	[14]
PM6:BTP-eC9	27.58	0.859	77.34	18.32	[15]
PM6:Y6	26.79	0.853	80.20	18.33	[16]
PM6:EH-HD-4F	27.5	0.84	79.3	18.38	[17]
PM6:BTP-eC9:$PC_{71}BM$	27.10	0.864	78.6	18.4	[18]
D18-Cl:N3	27.18	0.860	78.8	18.42	[19]
PM6:BTP-eC9:BTP-F	26.99	0..858	79.7	18.45	[20]
PM6:L8-BO	25.9	0.89	80.2	18.5	[21]
PBDB-TF:BTP-eC9	26.6	0.866	80.3	18.5	[22]
D18-Cl:G19:Y6	27.36	0.871	77.72	18.53	[23]
PM6:BTP-eC9	27.12	0.848	80.79	18.58	[24]
PM6:L8-BO	26.03	0.893	80.0	18.60	[25]
PBDB-TF:PB2F:BTP-eC9	26.8	0.863	80.4	18.6	[26]
PM6:BTP-eC9:L8-BO-F	27.35	0.853	80.0	18.66	[27]
D18-Cl:N3:$PC_{61}BM$	28.22	0.849	78.0	18.69	[28]
D18-Cl:L8-BO	26.6	0.922	75.6	18.7	[29]
PM6:L8-BO	26.11	0.89	80.6	18.74	[30]
PM6:L8-BO	26.37	0.89	79.94	18.77	[31]
PM6:BTP-eC9:BTP-S9	27.50	0.861	79.34	18.8	[32]
PBDB-TF:HDO-4Cl:eC9	27.05	0.866	80.51	18.86	[33]
PTQ10:m-BTP-PhC6 :$PC_{71}BM$	26.99	0.869	80.6	18.89	[34]
PBQx-TF:eC9-2Cl:F-BTA3	26.7	0.879	80.9	19.0	[35]
PTQ10:BTP-FTh:IDIC	27.17	0.87	80.6	19.05	[36]
PM1:L8-BO:BTP-2F2Cl	27.15	0.881	80.14	19.17	[37]
PM6:L8-BO:BTP-H2	26.68	0.892	80.7	19.2	[38]
**PM6:D18-Cl:L8-BO**	**26.66**	**0.91**	**79.24**	**19.22**	**This work**
PM6:D18:L8-BO	26.7	0.896	81.9	19.6	[39]

Table S4 The J_{ph}s and the ratios of the BOSCs and optimized TOSCs under different conditions.

Active layer	J_{ph}* (mA cm^{-2})	$J_{ph}$$^{\#}$ (mA cm^{-2})	J_{sat} (mA cm^{-2})	J_{ph}*/J_{sat} (%)	$J_{ph}$$^{\#}$/$J_{sat}$ (%)
PM6:L8-BO	26.35	24.42	27.24	96.73	89.64
PM6:D18-Cl:L8-BO	26.66	24.88	27.50	96.94	90.47
D18-Cl:L8-BO	25.38	22.26	27.10	93.65	82.14

Table S5. The hole mobility (μ_h), electron mobility (μ_e) values of the active layers with various D18-Cl content.

Active layer	μ_h (cm^2 V^{-1} s^{-1})	μ_e (cm^2 V^{-1} s^{-1})	μ_h/μ_e
PM6:L8-BO	8.52×10^{-4}	5.82×10^{-4}	1.46
PM6:D18-Cl:L8-BO	9.63×10^{-4}	9.47×10^{-4}	1.02
D18-Cl:L8-BO	7.70×10^{-4}	4.36×10^{-4}	1.77

Table S6. Morphology parameters extracted from the GIWAXS measurements of blend films with various D18-Cl content.

PM6:D18-Cl:L8-BO (wt:wt:wt)	FWHM of (100) Peak ($Å^{-1}$)	CCL of (100) Stacking (Å)	FWHM of (010) Peak ($Å^{-1}$)	CCL of (010) Stacking (Å)
1:0:1.2	0.088	71.4	0.391	16.0
0.8:0.2:1.2	0.086	73.1	0.375	16.8
0.7:0.3:1.2	0.084	74.8	0.373	16.9
0.6:0.4:1.2	0.090	69.8	0.379	16.6
0.5:0.5:1.2	0.096	65.5	0.381	16.5
0:1:1.2	0.098	64.1	0.391	16.0

4. References

[1] F. Liu, L. Zhou, W. Liu, Z. Zhou, Q. Yue, W. Zheng, R. Sun, W. Liu, S. Xu, H. Fan, L. Feng, Y. Yi, W. Zhang, X. Zhu, *Adv. Mater.* **2021**, 33, 2100830.

[2] X. Chen, D. Wang, Z. Wang, Y. Li, H. Zhu, X. Lu, W. Chen, H. Qiu, Q. Zhang, *Chem. Eng. J.* **2021**, 424, 130397.

[3] Y. Lin, Y. Firdaus, F. H. Isikgor, M. I. Nugraha, E. Yengel, G. T. Harrison, R. Hallani, A. El-Labban, H. Faber, C. Ma, X. Zheng, A. Subbiah, C. T. Howells, O. M. Bakr, I. McCulloch, S. D. Wolf, L. Tsetseris, T. D. Anthopoulos, *ACS Energy Lett.* **2020**, 5, 2935.

[4] M. Zhang, L. Zhu, G. Zhou, T. Hao, C. Qiu, Z. Zhao, Q. Hu, B. W. Larson, H. Zhu, Z. Ma, Z. Tang, W. Feng, Y. Zhang, T. P. Russell, F. Liu, *Nat. Commun.* **2021**, 12, 309.

[5] W. Feng, S. Wu, H. Chen, L. Meng, F. Huang, H. Liang, J. Zhang, Z. Wei, X. Wan, C. Li, Z. Yao, Y. Chen, *Adv. Energy Mater.* **2022**, 12, 2104060.

[6] M. Guan, W. Tao, L. Xu, Y. Qin, J. Zhang, S. Tan, M. Huang, B. Zhao, *J. Mater. Chem. A* **2022**, 10, 9746.

[7] Y. Zeng, D. Li, H. Wu, Z. Chen, S. Leng, T. Hao, S. Xiong, Q. Xue, Z. Ma, H. Zhu, Q. Bao, *Adv. Funct. Mater.* **2021**, 32, 2110743.

[8] J. Chen, J. Cao, L. Liu, L. Xie, H. Zhou, J. Zhang, K. Zhang, M. Xiao, F. Huang, *Adv. Funct. Mater.* **2022**, 32, 2200629.

[9] L. Wang, C. Guo, X. Zhang, S. Cheng, D. Li, J. Cai, C. Chen, Y. Fu, J. Zhou, H. Qin, D. Liu, T.

Wang, *Chem. Mater.* **2021**, 33, 8854.
[10] Q. Liu, Y. Jiang, K. Jin, J. Qin, J. Xu, W. Li, J. Xiong, J. Liu, Z. Xiao, K. Sun, S. Yang, X. Zhang, L. Ding, *Science Bulletin* **2020**, 65, 272.
[11] J. Zhang, F. Bai, I. Angunawela, X. Xu, S. Luo, C. Li, G. Chai, H. Yu, Y. Chen, H. Hu, Z. Ma, H. Ade, H. Yan, *Adv. Energy Mater.* **2021**, 11, 2102596.
[12] X. Zhang, C. Li, J. Xu, R. Wang, J. Song, H. Zhang, Y. Li, Y.-N. Jing, S. Li, G. Wu, J. Zhou, X. Li, Y. Zhang, X. Li, J. Zhang, C. Zhang, H. Zhou, Y. Sun, Y. Zhang, *Joule* **2022**, 6, 444.
[13] Y. Lin, M. I. Nugraha, Y. Firdaus, A. D. Scaccabarozzi, F. Aniés, A.-H. Emwas, E. Yengel, X. Zheng, J. Liu, W. Wahyudi, E. Yarali, H. Faber, O. M. Bakr, L. Tsetseris, M. Heeney, T. D. Anthopoulos, *ACS Energy Lett.* **2020**, 5, 3663.
[14] C. Li, J. Zhou, J. Song, J. Xu, H. Zhang, X. Zhang, J. Guo, L. Zhu, D. Wei, G. Han, J. Min, Y. Zhang, Z. Xie, Y. Yi, H. Yan, F. Gao, F. Liu, Y. Sun, *Nat. Energy* **2021**, 6, 605.
[15] Y. Cheng, B. Huang, X. Huang, L. Zhang, S. Kim, Q. Xie, C. Liu, T. Heumuller, Z. Liu, Y. Zhang, F. Wu, C. Yang, C. J. Brabec, Y. Chen, L. Chen, *Angew. Chem., Int. Ed.* **2022**, 61, 202200329.
[16] D. Li, F. Geng, T. Hao, Z. Chen, H. Wu, Z. Ma, Q. Xue, L. Lin, R. Huang, S. Leng, B. Hu, X. Liu, J. Wang, H. Zhu, M. Lv, L. Ding, M. Fahlman, Q. Bao, Y. Li, *Nano Energy* **2022**, 96, 107133.
[17] S. Chen, L. Feng, T. Jia, J. Jing, Z. Hu, K. Zhang, F. Huang, *Sci. China Chem.* **2021**, 64, 1192.
[18] Y. Lin, A. Magomedov, Y. Firdaus, D. Kaltsas, A. El-Labban, H. Faber, D. R. Naphade, E. Yengel, X. Zheng, E. Yarali, N. Chaturvedi, K. Loganathan, D. Gkeka, S. H. AlShammari, O. M. Bakr, F. Laquai, L. Tsetseris, V. Getautis, T. D. Anthopoulos, *ChemSusChem* **2021**, 14, 3569.
[19] J. Qin, Q. Yang, J. Oh, S. Chen, G. O. Odunmbaku, N. A. N. Ouedraogo, C. Yang, K. Sun, S. Lu, *Adv. Sci.* **2022**, 9, 2105347.
[20] Y. Li, Y. Cai, Y. Xie, J. Song, H. Wu, Z. Tang, J. Zhang, F. Huang, Y. Sun, *Energy Environ. Sci.* **2021**, 14, 5009.
[21] X. Song, K. Zhang, R. Guo, K. Sun, Z. Zhou, S. Huang, L. Huber, M. Reus, J. Zhou, M. Schwartzkopf, S. V. Roth, W. Liu, Y. Liu, W. Zhu, P. Muller-Buschbaum, *Adv. Mater.* **2022**, 34, 2200907.
[22] L. Hong, H. Yao, Y. Cui, P. Bi, T. Zhang, Y. Cheng, Y. Zu, J. Qin, R. Yu, Z. Ge, J. Hou, *Adv. Mater.* **2021**, 33.
[23] Z. Chen, W. Song, K. Yu, J. Ge, J. Zhang, L. Xie, R. Peng, Z. Ge, *Joule* **2021**, 5, 2395.
[24] X. Xiong, X. Xue, M. Zhang, T. Hao, Z. Han, Y. Sun, Y. Zhang, F. Liu, S. Pei, L. Zhu, *ACS Energy Lett.* **2021**, 6, 3582.
[25] J. Song, L. Zhu, C. Li, J. Xu, H. Wu, X. Zhang, Y. Zhang, Z. Tang, F. Liu, Y. Sun, *Matter* **2021**, 4, 2542.
[26] T. Zhang, C. An, P. Bi, Q. Lv, J. Qin, L. Hong, Y. Cui, S. Zhang, J. Hou, *Adv. Energy Mater.* **2021**, 11, 2101705.
[27] Y. Cai, Y. Li, R. Wang, H. Wu, Z. Chen, J. Zhang, Z. Ma, X. Hao, Y. Zhao, C. Zhang, F. Huang, Y. Sun, *Adv. Mater.* **2021**, 33, 2101733.
[28] K. Jin, Z. Xiao, L. Ding, *J. Semicond.* **2021**, 42, pages433.
[29] G. Cai, Z. Chen, X. Xia, Y. Li, J. Wang, H. Liu, P. Sun, C. Li, R. Ma, Y. Zhou, W. Chi, J. Zhang, H. Zhu, J. Xu, H. Yan, X. Zhan, X. Lu, *Adv. Sci.* **2022**, 9, 2200578.
[30] X. Xu, L. Yu, H. Meng, L. Dai, H. Yan, R. Li, Q. Peng, *Adv. Funct. Mater.* **2021**, 32, 2108797.
[31] H. Meng, C. Liao, M. Deng, X. Xu, L. Yu, Q. Peng, *Angew. Chem., Int. Ed.* **2021**, 60, 22554.
[32] L. Zhan, S. Li, Y. Li, R. Sun, J. Min, Z. Bi, W. Ma, Z. Chen, G. Zhou, H. Zhu, M. Shi, L. Zuo, H.

Chen, *Joule* **2022**, 6, 662.
[33] P. Bi, S. Zhang, Z. Chen, Y. Xu, Y. Cui, T. Zhang, J. Ren, J. Qin, L. Hong, X. Hao, J. Hou, *Joule* **2021**, 5, 2408.
[34] S. Bao, H. Yang, H. Fan, J. Zhang, Z. Wei, C. Cui, Y. Li, *Adv. Mater.* **2021**, 33, e2105301.
[35] Y. Cui, Y. Xu, H. Yao, P. Bi, L. Hong, J. Zhang, Y. Zu, T. Zhang, J. Qin, J. Ren, Z. Chen, C. He, X. Hao, Z. Wei, J. Hou, *Adv. Mater.* **2021**, 33, 2102420.
[36] K. Chong, X. Xu, H. Meng, J. Xue, L. Yu, W. Ma, Q. Peng, *Adv. Mater.* **2022**, 34, 2109516.
[37] R. Sun, Y. Wu, X. Yang, Y. Gao, Z. Chen, K. Li, J. Qiao, T. Wang, J. Guo, C. Liu, X. Hao, H. Zhu, J. Min, *Adv Mater* **2022**, 2110147.
[38] C. He, Y. Pan, Y. Ouyang, Q. Shen, Y. Gao, K. Yan, J. Fang, Y. Chen, C. Ma, J. Min, C. Zhang, L. Zuo, H. Chen, *Energy Environ. Sci.* **2022**, DOI:10.1039/D2EE00595F.
[39] L. Zhu, M. Zhang, J. Xu, C. Li, J. Yan, G. Zhou, W. Zhong, T. Hao, J. Song, X. Xue, Z. Zhou, R. Zeng, H. Zhu, C. C. Chen, R. C. I. MacKenzie, Y. Zou, J. Nelson, Y. Zhang, Y. Sun, F. Liu, *Nat Mater* **2022**, 22, 2060.
